# Knowledge about Hand Hygiene and Related Infectious Disease Awareness among Primary School Children in Germany †

**DOI:** 10.3390/children9020190

**Published:** 2022-02-02

**Authors:** Kristin Klar, Dennis Knaack, Stefanie Kampmeier, Anna Katharina Hein, Dennis Görlich, Siegfried Steltenkamp, Ulrike Weyland, Karsten Becker

**Affiliations:** 1 Institute of Medical Microbiology, University Hospital Münster, 48149 Münster, Germany; kklar@uni-muenster.de (K.K.); dennis.knaack@sfh-muenster.de (D.K.); 2 Institute of Hygiene, University Hospital Münster, 48149 Münster, Germany; stefanie.kampmeier@ukmuenster.de; 3 Institute of Education, Westphalian Wilhelms-University of Münster, 48143 Münster, Germany; akhein@uni-muenster.de (A.K.H.); ulrike.weyland@uni-muenster.de (U.W.); 4 Institute of Biostatistics and Clinical Research, Westphalian Wilhelms-University of Münster, 48149 Münster, Germany; dennis.goerlich@ukmuenster.de; 5 Ophardt Hygiene-Technik GmbH + Co. KG, 47661 Issum, Germany; ssteltenkamp@ophardt.com; 6 Friedrich Loeffler-Institute of Medical Microbiology, University Medicine Greifswald, 17475 Greifswald, Germany

**Keywords:** hand hygiene, infectious disease, sanitary facilities, primary school children, infection prevention, questionnaire, Germany, North Rhine–Westphalia

## Abstract

Hand hygiene is a cornerstone of infection prevention. However, few data are available for school children on their knowledge of infectious diseases and their prevention. The aim of the study was to develop and apply a standardized questionnaire for children when visiting primary schools to survey their knowledge about infectious diseases, pathogen transmission and prevention measures. Enrolling thirteen German primary schools, 493 questionnaires for grade three primary school children were included for further analyses, comprising 257 (52.1%) girls and 236 (47.9%) boys with an age range of 8–11 years. Out of 489 children, 91.2% participants indicated that they knew about human-to-human transmissible diseases. Of these, 445 children responded in detail, most frequently mentioning respiratory and gastrointestinal diseases, followed by childhood diseases. Addressing putative hygiene awareness-influencing factors, it was worrisome that more than 40.0% of the children avoided visiting the sanitary facilities at school. Most of the children (82.9%) noted that they did not like to use the sanitary facilities at school because of their uncleanliness and the poor hygienic behavior of their classmates. In conclusion, basic infection awareness exists already in primary school age children. Ideas about the origin and prevention of infections are retrievable, however, this knowledge is not always accurate and adequately contextualized. Since the condition of sanitary facilities has a strong influence on usage behavior, the child’s perspective should be given more consideration in the design and maintenance of sanitary facilities.

## 1. Introduction

Hand hygiene is generally accepted as the primary preventive measure for the reduction of infectious diseases [1] and a multitude of publications have investigated various facets of hand hygiene, especially in the health care sector [2,3,4]. However, key hygienic skills, particularly hand hygiene rules, are also essential for the general population, especially for situations in which there is a possibility of transmission and ingestion of pathogens in the home and in communal environments (e.g., food preparation, use of sanitary facilities). Despite the achievements of the last decades, compliance and proper execution of hygiene practices are of unabated interest even in developed countries. The reasons for this are (i) the emergence of novel pathogens as currently experienced with SARS-CoV-2 [5], (ii) the selection and global spread of multidrug-resistant organisms (MDROs) [6,7,8,9,10], (iii) the medical progress characterized by the increased application of immune system-impairing procedures and colonization-vulnerable devices [11,12,13,14,15,16], (iv) the demographic changes leading to higher susceptibility of subpopulations to infectious diseases, such as the increase of older and multi-morbid individuals [17,18,19] and (v) the migration and lifestyle aspects leading to the transmission and distribution of MDROs [20,21,22]. Emerging clonal lineages of notorious MDROs with changed epidemiology and host spectra (e.g., community- and livestock-associated MRSA and hypervirulent, multi-resistant *Klebsiella pneumoniae* lineages) also add to the burden of diseases, necessitating health behavior programs [6,9,23,24,25,26,27,28,29,30].

Primary school children are in a stage of development in which basic life and behavioral skills are developing. It has been shown that health behavior-forming programs in the school setting have a significant impact on later life [28]. In Germany, various health programs are run for primary schools, especially with regard to healthy nutrition inside and outside school and physical activity exercises (for more information, consider, e.g., Bundeszentrum für Ernährung Home page. Available online: https://www.bzfe.de/bildung/lernort-schule-und-kita (accessed on 3 January 2022)). Health and prevention programs can be particularly promising if integrated into the education system at an early stage [28,31,32,33]. With regard to acute illnesses, children and adolescents in Germany up to the age of seventeen most frequently suffer from colds and flu-like infections (respiratory infections) followed by gastrointestinal infections, as recorded by the German Health Interview and Examination Survey for Children and Adolescents (KiGGS) [34]. Besides aspects of morbidity, infectious diseases often lead to days of absence from school. However, the effect of infectious diseases is not limited to absenteeism; they also affect the child’s learning progress. Moreover, other factors should be taken into account, such as care times of the child by the parents, resulting in absence from work [35]. A direct link for this effect has been shown for seasonal influenza [36].

Up to now, little research has been performed on the knowledge of children visiting primary schools about infectious diseases, the causes of infection and pathogen transmission as well as on knowledge and skills on respective prevention measures [37]. In particular, there are only a few data sets available on the hand hygiene of school children at this level. Related (pilot) studies include hand hygiene in the context of absenteeism from school and interventions to improve hand hygiene, focusing on proper and mandatory handwashing [37,38,39,40,41,42,43,44]. A pan-European antibiotic and hygiene teaching resource (e-Bug) was established a decade ago based on the results of a questionnaire investigating the educational structures, educational resources and hygiene-related campaigns of European schools [45]. It was concluded that too little was being done across Europe to educate school children on the importance of appropriate antibiotic use and antibiotic resistance [45]. Nevertheless, the current state of research is unsatisfactory and there is a need to obtain more detailed research data on hand hygiene in primary school-aged children. Thus, in this exploratory study, a pre-tested written questionnaire for primary school children from the third grade upwards was developed and implemented in thirteen participating primary schools, and the collected data were comprehensively analyzed. The study aimed (i) to obtain reliable data on the knowledge of the target group of children about hand hygiene, (ii) to raise awareness of this issue among teachers, school authorities, parents and other stakeholders and (iii) to obtain baseline data for follow-up studies and interventions.

## 2. Materials and Methods

### 2.1. Participants

**Figure 1:**
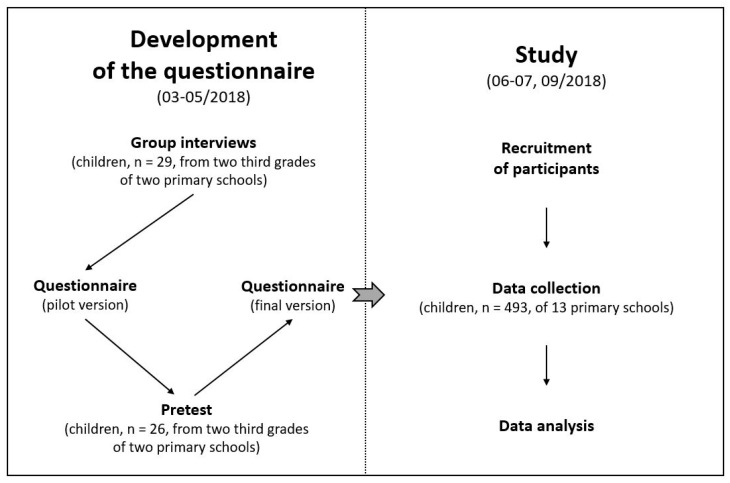
Study design and flowchart of the development of the questionnaire.

This study was part of the INTERREG VA-funded joint projects “EurHealth-1Health” and “health-i-care”. Thus, school children enrolled at visited primary schools in the county of Steinfurt (German federal state of North Rhine–Westphalia), which is part of the Dutch–German EUREGIO region. The study design is depicted in Figure 1. The final questionnaire was implemented in thirteen primary schools based on group interviews and pretests at two other schools in the county. The fifteen participating primary schools are located in 10 out of 24 municipalities in the county of Steinfurt (Table A1). The number of inhabitants in these 10 municipalities ranged between approximately 6600 and 37,800 (Landesbetrieb IT.NRW Home Page. Available online: https://www.it.nrw/statistik (accessed on 31 December 2019)).

At the beginning of the 2017/2018 school year, the total number of school children of the thirteen primary schools was approximately 2600, with a range of 94–340 school children per primary school. In grade three, the number of school children was between 25 and 76. The number of the sanitary facilities for school children varied between 2 and 8 (Table A2).

The questionnaire was designed for third-grade pupils of primary schools. A consent form signed by their parents or guardians allowing them to participate was a prerequisite. In total, the questionnaire was filled out anonymously by 494 third-graders. Since one completed questionnaire was classified as invalid due to inadequate processing, 493 valid cases were included for further analyses, which represents a response rate of 76.4%.

### 2.2. Development of the Questionnaire

A standardized written questionnaire in German was developed in several steps, including (i) group interviews as a basis for the elaboration of a (ii) pretest version followed by the development of the (iii) final questionnaire (Figure 1). For the development of the questionnaire, two additional primary schools participated.

Third-graders (8–10 years old) of two primary schools were interviewed anonymously on two days in March 2018 in small groups (6–9 school children per group), focusing on their knowledge about pathogens and infectious diseases, including transmission and prevention aspects. Overall, 29 school children participated in the group interviews (girls, *n* = 15 (51.7%); boys, *n* = 14 (48.3%)). The results of the group interviews were considered for the development of the questionnaire’s pilot version. This version was pretested by 26 school children (girls, *n* = 14 (53.8%); boys, *n* = 12 (46.2%); 8–10 years old) out of two third-grade classes at the end of May 2018 to find out whether the questionnaire corresponds to the requirement level of the target group and meets the time limits. Pretesting led to changes in terms of, e.g., layout adaptation and wording.

The final questionnaire contained sixteen questions partially divided into sub-questions. The (sub-)questions were open (*n* = 1), half-open (*n* = 5) and closed (*n* = 15). The questionnaire was divided into two parts: (i) knowledge about hand hygiene and (ii) health maintenance and health protection. The last page of the questionnaire was reserved for the collection of demographic data.

### 2.3. Data Collection and Analyses

The data collection applying the final questionnaire was conducted within approximately five weeks from 14 June to 10 July in 2018, except for one school that participated later in September 2018 due to renovations (Figure 1). Processing of the questionnaire by the school children was timed for one school lesson (45 min). Data collection was guided step by step in each case by the same study advisor (a trained teacher). Briefly, the questionnaire was projected on the wall using an overhead projector or a white board, respectively, so that the school children could read along and know which question was to be answered. In addition, the questions were read aloud. To support concentration on the question being worked on and not to skip ahead, the school children were given a sheet of paper to cover the other parts of the questionnaire. The primary schools could decide for themselves whether a teacher would be present during data collection and whether a partition would be set up between the school children. In the pretest, both options were tried out. The primary schools could make their own decision on the teaching structure depending on the forms of learning normally preferred (e.g., cooperative forms of learning). Assistance for school children with special needs was admitted.

Statistical analyses (Pearson’s chi-squared test, Fisher’s exact test, McNemar’s test and Wilcoxon test) and data evaluation were conducted with the Statistical Package for the Social Sciences (SPSS) version 26 and 27 (IBM, Armonk, New York, NY, USA). Statistical significance was considered at *p* ≤ 0.05.

## 3. Results and Discussion

For analyses, 493 valid cases of accomplished questionnaires of third-grade school children were included. The gender distribution was 257 (52.1%) girls and 236 (47.9%) boys. School children were between 8–11 years old (median, 9 years old). An age distribution by gender is shown in Table A3. Of the 493 questionnaires (valid cases), 57 questionnaires (≈11.6%) were collected later in September 2018 due to renovations at one school.

The aim of the first part of the questionnaire was to identify how often school children visited the sanitary facilities at school, how they judged the sanitary facilities at school and what impact this had on the judgement of their behavior. They were also asked about the importance they placed on hand hygiene in relation to the use of sanitary facilities at school.

At the beginning of the questionnaire, the school children had the opportunity to assign school grades (1, very good; 2, good; 3, satisfactory; 4, sufficient; 5, poor; and 6, deficient) to the sanitary facilities of their school. Most school children (30.3%; *n* = 149/492 valid cases) rated the sanitary facilities with the school grade 3 (mean value, 3.38) (Table 1). Of note, close to half (43.9%) of the school children chose a school grade between 4 (sufficient) and 6 (deficient). Consistently, a similar percentage of children (44.8%) indicated that they would prefer not to use the toilet during school by choosing the response option “Mostly not at all”. The distribution of girls and boys who gave this answer was almost equal (girls, *n* = 112 (23.0%); boys, *n* = 106 (21.8%); 218/487 valid cases). “I visit the toilet at school more than three times” per day was the response of only 23 school children (4.7%) (Table 2). Lower school children from Sweden gave a median of “3” in the evaluation of school toilets (1, terrible; 5, excellent) [46]. A parent survey conducted in Giessen (Germany) asked whether their children used the sanitary facilities at school [47]. Similar to our study, children´s avoidance behavior was evident in relation to toileting at school. In addition, over 75.0% of parents indicated that children predominantly use the sanitary facilities to urinate only, but less for bowel movements [47]. Swedish school children also stated that the sanitary facilities at school were more likely used for urinating [46].

To analyze the behavior in terms of toilet visiting in more detail, the respective reasons were queried (“Why do I like/Why do I don’t like to go to the sanitary facilities at school?”). Out of 493 school children, 79.7% (*n* = 393) replied that they didn’t like using the sanitary facilities at school. No significant gender disparities were noted (girls, *n* = 208 (42.2%); boys, *n* = 185 (37.5%)). Only 44 girls and 37 boys (81 school children; 16.4%) said “Yes, I like going to the toilet at school” and 3.9% (19 school children) didn’t answer the question.

To investigate school children´s motivation for visiting the sanitary facilities at school, the question was offered with free text input (multiple answers were possible). The majority of the school children (*n* = 400) responded with the sentence “No, I don’t like to go to the sanitary facilities at school, because…”. The most common reasons for not using the sanitary facilities at school were the uncleanliness of the sanitary facilities and the (mis-)behavior of classmates (Table 3). An interview study with school children aged 9–16 from Sweden reported similar results [48]. The children described the sanitary facilities as unclean and named general conditions, such as the smell. Other similarities were that children also reported being disturbed by classmates or that it was uncomfortable using the sanitary facilities at school. In contrast to our study, the school children from Sweden indicated that they forgot the need to go to the toilet during break, e.g., they would rather play. As a result, the children wanted to go to the toilet during the lesson and also found it less stressful because all the other children were in class [48]. The time factor and the sanitary facilities were also mentioned by the focus groups (school children 6–11 years old) from another study performed in England [49]. In addition, some children indicated that they do not go to the toilet during lesson because they might miss the content of the lesson (Table 3). In an Australian study, school children (6–10 years old) referred to misbehavior or disruptions by other school children in connection with the use of the sanitary facilities [50].

Of the 84 answers beginning “Yes, I like to go to the sanitary facilities at school, because…”, 11 answers were not informative or evaluable. Overall, 34 school children gave the answer that they felt “an urgent need to visit the school toilet”. The hygiene of sanitary facilities was the second most common answer (*n* = 28). Further answers can be categorized as follows: general time off or time off from class (*n* = 5), orientation to the need (hand hygiene) (*n* = 5), the atmosphere of the sanitary facilities (*n* = 5), the equipment and function of the sanitary facilities (*n* = 3), the reduction of microbial pathogens (*n* = 1) and behavior/need of classmates (*n* = 1).

A further question concerned how often children practice hand hygiene before or after certain situations and whether there is a difference between the situation at school and at home? Table 4 and Table 5 list the frequencies with which school children specified that they wash their hands before or after certain situations or actions. Of particular interest are the results for statements 2, 4 and 6 (Table 4) and statements 3, 5, 6 and 10 (Table 5).

The statement “I wash my hands at school before eating, e.g., bread during the break or lunch” was affirmed by slightly less than half of the school children (48.0%); however, there was variation in terms of frequency between 23.7% (always) and 24.3% (often). Approximately one quarter completely (24.5%) and another quarter predominantly (27.4%) denied hand washing prior to eating. While children are taught to “wash their hands before eating” prior to school enrollment in day care centers, about half of the children do not practice this at school. This is in contrast to the situation at home, where significantly more, i.e., about three-quarters of the school children (77.4%, *p* < 0.001) answered that they washed their hands always or, at least, often (Table 5; statement 3). Thus, knowledge of hand washing before eating is already developed and it is also practiced by the majority at this age. Hence, other conditions may compromise their implementation at school. That only one sink per classroom is provided may contribute to this discrepancy, and the answers of the school children (keyword: time factor) as well as the proportion of average class sizes at the participating schools (*n* = 22.7) support this hypothesis. Moreover, if school children want to overcome this situation by using the primary school sanitary facilities, they find themselves in the dilemma between the one-sink situation of their classroom and the negative connotation of the toilet environment. Thus, avoidance behavior is provoked, as has been shown, also, by others.

The extent to which the sanitary facilities (sinks and toilets) are used by school children depends on their condition [35]. School children aged 9–11 years in England and Sweden perceived the sanitary facilities at school as unclean, stinky and unpleasant (likewise in relation to disturbances by other children) [51]. A study that focused on the conditions of hygiene in schools revealed that the sinks in primary schools were better equipped compared to other types of school. However, the sinks in the classrooms were less well equipped than the sinks in the sanitary facilities. It was also evident that classroom sinks were used less by children to wash their hands and more frequently, for example, by teachers to rinse the blackboard sponge [52]. Thus, rethinking of the importance and usage of sinks in classrooms and their value for hand hygiene to prevent the development and transmission of infections is necessary. However, other uses (e.g., washing utensils used in art lessons) do not necessarily have to be excluded.

Avoidance of using the sanitary facilities at school may have direct negative impacts for the children, e.g., constipation and urinary tract infections [51]. In addition, this avoidance behavior may also have an indirect negative effect on the children’s goal of washing hands only at the sanitary facilities (e.g., before eating and after the break), which is important for infection prevention.

An even worse situation became apparent when the answers to the statement “I wash my hands at school after the break” were analyzed (Table 4; statement 6). The majority of the school children indicated that they “never” or “seldom” wash their hands (73.2%). In contrast, in the home setting (Table 5; statement 6), 36.4% of the school children answered that they wash their hands “never” or “seldom” after playing outside. The results of both statements show a difference of 36.8%. Between the results of both statements is a significant difference (*p* < 0.001) that indicates that there is a change in children’s behavior between school and home. The reasons for this may be due to more intensive observation at home and/or the much better conditions for hygiene procedures at home. In connection with the results for statement 10 (Table 5) regarding hand washing when coming home, it would be interesting to know whether the distribution of answers would be different if the questionnaire were conducted today in light of the COVID-19 pandemic.

Of particular interest were the answers to the question: “How often do I wash my hands after going to the toilet?” (Table 4). For 76.7% of the school children, the answer was “always” at school and only 1.2% of the children indicated that they never wash their hands in this situation. This percentage is not statistically significantly different (*p* = 0.396; Wilcoxon test; Z-score = −0.849) from the responses related to hand hygiene after toileting at home (“always”, 75.7%; “never”, 1.0%) (Table 5). The answers to another question confirmed relatively well-developed hygiene awareness of handwashing after toilet use. The school children were asked to write a rationale (free text) regarding why hand hygiene after toilet use is important for them. Multiple answers were possible. The responses indicated that washing hands after going to the toilet is important for most school children (98.5%; 451/458 valid cases). The most given reason was related to the notion of “pathogens”. The children noted that pathogens are, e.g., on the hands or on the sanitary facilities (e.g., toilet). A further 115 answers of the school children focused thematically on the development of infections. Thus, with a certain degree of probability, these results indicated that school children at this age already have knowledge of the importance of practicing hand hygiene in order to maintain individual and collective health. Furthermore, it is noticeable that school children in primary schools have the knowledge that bacteria exist on their hands. However, the existence of other kinds of pathogens (e.g., viruses or parasites) as well as an idea of pathogenic (“bad”) versus non-pathogenic, commensal or even mutualistic (“good”) bacteria as part of the human microbiota (“physiological flora”) is unknown at this age. The knowledge that hand hygiene is important to remove microbial pathogens was also supported by the results for focus groups (children aged 6–11 years) in England [49].

**Figure A1:**
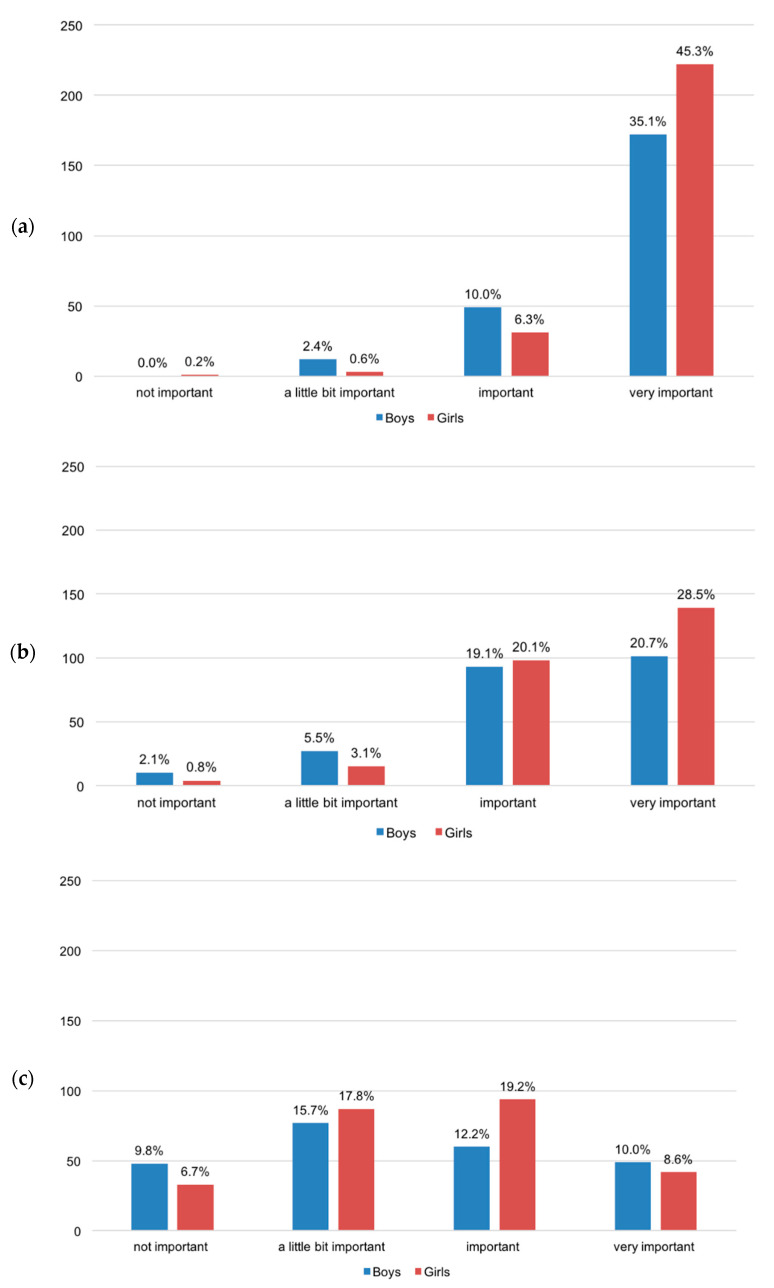
Bar charts (**a**–**c**) illustrate the importance of handwashing for school children during a visit to sanitary facilities (**a**) after bowel movement (*n* = 490), (**b**) urination (*n* = 487) and (**c**) independently of the visiting purpose (*n* = 490), respectively.

Since awareness may differ for bowel movement and urination procedures, particularly in the case of boys, this respective question was included. Over 95.0% of the school children found it “important” or “very important” to wash their hands after a bowel movement. Stratified by gender, 51.6% of the girls and significantly less boys (45.1%) affirmed this statement (Figure A1a) *p* = 0.025; chi-squared test). For only 3.3% of the school children, it was “not important” or only a “little bit important” to wash their hands after a bowel movement. In the case of urination (Figure A1b), 431 (88.5%) school children considered that it is “important” or “very important” to wash the hands thereafter. Statistical verification shows that the responses “important” or “very important” differed significantly by gender (*p* = 0.003; chi-squared test). Around 11.5% of the school children perceive hand hygiene as “not important” or a “little bit important” in this case. That is a percentage increase of 8.2% in comparison to the bowel movement results. The sanitary facilities of all participating schools had urinals in the toilet rooms of the boys. Here, it would be of interest to determine the boys’ views on hand hygiene when they would use either the toilet or the urinal to urinate.

School children may visit the school sanitary facilities not only for the actual purpose of use. Depending on their location (e.g., outside the main school building next to the playground) they may use the sanitary facilities to play (e.g., hide-and-seek) or to escape supervision. Within the questionnaire, two examples (sanitary facilities as a “hiding place” and as offering an opportunity to share secrets) were given so that the school children have an idea what is meant by the question. As visible in Figure A1c, it is “important” or “very important” for 50.0% of the school children (*n* = 245) to wash hands regardless of the reason for visiting the toilets. Exactly the same number of school children answered “not important” or a “little bit important”.

Pairwise comparison of the results concerning the importance of hand hygiene revealed significant results for “bowel movement” vs. “urination” (*p* < 0.001; Wilcoxon test; Z-score = −12.904), “bowel movement” vs. “visiting toilets for other reasons” (*p* < 0.001; Wilcoxon test; Z-score = −16.776) and “urination” vs. “visiting toilets for other reasons” (*p* < 0.001; Wilcoxon test; Z-score = −14.159). Thus, a tendency was observed that bowel movement received the highest awareness concerning hand washing, followed by urination and visiting toilets for other reasons.

**Figure 2:**
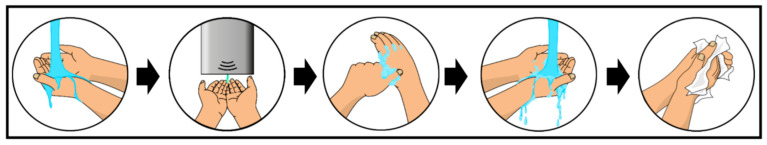
Hand hygiene steps (Question 9 of the questionnaire).

A further point of the questionnaire addressed the practical hand washing skills of the school children. They were asked to sort the displayed pictures of hand hygiene steps in the right order by adding the numbers one to five to the pictures (Figure 2). Knowing the correct sequence of hand washing steps is important to facilitate the practical teaching of effective hand hygiene in children [49]. A total of 385 school children (78.1%) were able to sort the hand hygiene steps correctly. Of the 108 school children (21.9%) who weren’t able to sort the order correctly, 45 (41.7%) of the school children started the hand hygiene steps with picture two “Pull the soap out of the soap dispenser” instead of “Get your hands wet”, and 86 school children (79.6%) selected as the second hand hygiene step “Get your hands wet” instead of “Pull the soap out of the soap dispenser”. Overall, the answers of 59 school children included more than one mistake. Four-hundred-and-eighty-five school children (98.4%) correctly selected “Dry hands well” (as step five).

The second part of the questionnaire dealt with questions about the maintenance of health and protection against diseases. The majority of the school children (*n* = 439/491 valid cases; 89.4%) ticked the answer option “always” or “often” when asked about the statement “I wash my hands several times a day” as a possible measure to maintain individual health. About 10.6% of the school children chose the answer option “rarely” or “never” and in this case significantly more boys (*n* = 36) as opposed to 16 girls (*p* = 0.001/chi-squared test).

The question “How can you protect yourself against disease?” comprised seven statements, for which the school children were asked to answer with “right” or “false” (Table 6). The statements 1–7 were answered correctly by more than 80.0% of the school children. Of particular relevance is the fact that 99.4% of the school children (489/492 valid cases) responded correctly to the statement “I wash my hands with soap.” Regarding statement 1 (“I can be vaccinated against specific diseases.”), it should be noted that school children may have answered incorrectly (5.1%) for several reasons, e.g., the school children may know that they have not been vaccinated against certain diseases or they may know that their parents have objections to vaccination. Covering an open wound with a plaster or bandage to protect against disease (statement 3) was considered “right” by 450 school children (91.8%). Within the questionnaire survey, this statement was accompanied by the remark that it was meant to be an “open wound” and not a “slight skin abrasion”. However, for 40 school children (8.2%) this statement was false in connection with protection against disease, probably due to domestic experience of healing wounds without the protection of a sticking plaster. When analyzing the next statement “I keep distance from ill people who are suffering, e.g., from a cough or sniffles”, it is notable that 52 school children (10.6%) responded “false”. Presumably school children at this age keep less of a distance from people in their family environment (e.g., parents or siblings) if they are ill because of their special need for close physical contact at this age. Statement 6, asking about playing with the computer or watching TV as a “protective measure”, was answered in the right way by 412 school children (84.9%). The other 73 school children (15.1%), of which were significantly (*p* < 0.001) more boys (*n* = 58), ticked the wrong answer. However, this statement might be considered from the perspective that games on digital media are played alone and thus there is no contact with other individuals, which could be interpreted as implying protection against disease.

In order to obtain detailed information from the school children, open questions were developed that focused on three key aspects: human-to-human transmissible diseases, cause of diseases and transmission routes of diseases. Multiple answers were possible; thus, both correct and incorrect partial answers may appear concurrently.

First, 446 of a total of 489 school children indicated that they knew about human-to-human transmissible diseases; 445 written answers were noted, including wrong answers. Infections of the respiratory and gastrointestinal tract were listed most frequently, followed by childhood infections; other infectious diseases were mentioned sporadically (Table 7). In general, 63 wrong or partially wrong answers were given, e.g., cancer and allergies. Instead of diseases, symptoms of diseases were also mentioned, e.g., cough, sniffles, fever, vomit and diarrhea, that were not included in the wrong answers. Thus, school children at this level do not differentiate between infection and symptom(s). However, it was astonishing how many different disease entities (overall, *n* = 24) were mentioned by them.

Second, 58.4% of the school children responded that they know what can cause such transmissible diseases (affirmative answers, *n* = 281/481 valid cases). The related open question was completed by 291 school children (including wrong answers). While answers such as microbes, germs, bacteria, fungi, parasites and/or viruses were expected, only 41/291 (14.1%) school children responded as anticipated. Although only a few school children noted bacteria and/or viruses as a cause of a transmissible disease, these terms can already be part of the acquired vocabulary of school children at this level because they used these terms to answer other questions in this questionnaire (e.g., question about the importance of hand hygiene after using the toilet). Concerning the “incorrect” answers, it was interesting that 117 (40.2%) named an external factor, such as the situation of being outside with inadequate clothing, e.g., “Yes, if, for example, you walk around outside in winter without a scarf or cap” (girl, 8-year-old). Additionally, an inappropriate hygiene behavior was the content of 52 (17.9%) answers. The third and fourth most frequent “false” answers included symptoms of infections (*n* = 35; 12.0%) and ways of transmission of infections (*n* = 28; 9.6%).

Third, compared to 161 (34.3%) school children who gave a negative reply, 308 (65.7%) school children stated that they know how infections can be transmitted. While only 469 (valid cases) school children answered this yes/no question, a total of 332/490 (67.8%) school children filled in the subsequent answer field. The most mentioned infection route was transmission by droplet infection (*n* = 161, 48.5%), followed by smear infection (*n* = 133, 40.1%) and indirect transmission (*n* = 2, 0.6%). Other transmission routes of infections were not mentioned. Further answers or partial answers were declared as incorrect because there was no relation to putative transmission routes noting, for example, pathogens (*n* = 32), infections or symptoms (*n* = 3) and contamination (*n* = 19). Some comments have been categorized twice because the given comment could not be clearly assigned to one of the defined categories or because it addressed multiple categories.

Using exemplary statements that were intended to stimulate the imaginations of the participants, more data on the knowledge of school children about the transmission routes of pathogens were collected (Table 8). Here, school children were to imagine that they had a cough, sniffles or diarrhea and how they could contribute to avoid the transmission of those contagious diseases to fellow humans. The school children had the choice to answer whether the statement was right or false. Eighty-five percent of the school children answered all the statements correctly. A wrong answer to the statements was given for 10.0% or less of all statements, except for one statement (“I don’t spit on the floor.”) that was ticked by 73 school children (14.9%) incorrectly. In general, no significant differences between the answers of girls and boys can be noticed.

Overall, our study has the following limitations: Only primary schools of a defined administrative area were included to meet the EUREGIO affiliation but also to avoid influences of the educational structures in Germany, which are organized on the federal state level. A future German-wide or European study addressing this point is warranted. The participating primary schools were diverse in terms of various factors, such as school size and school location (urban or rural). Moreover, the teaching of and awareness of topics related to hygiene and infections in the broadest sense could differ between schools and classes. Further to this, it is to be mentioned that general hygiene behavior and awareness regarding infectious diseases, especially with respect to prevention measures, may be handled differently and have varying connotations in different families. In the survey form chosen in this study (standardized written questionnaire presented face-to-face) it should be noted that the interviewer (study advisor) and the respondents (school children) are in a complex interaction during the questioning. As a consequence, the interviewer’s behavior may have an influence on the respondents’ answers [53]. However, to limit this influence, the data collection was managed in our study by the same study advisor.

## 4. Conclusions

In summary, our study revealed that hand hygiene was an important measure for children of primary schools after visiting the toilet facilities. However, the importance of hand hygiene for the school children varied depending on the purpose of visiting the toilet. It was highest after bowel movement and decreased in its extent after urination and visiting the toilets for purposes other than those for which they were intended. We can conclude that the main objectives of school children for hand hygiene after going to the toilet were the removal of pathogens from their hands and the prevention of infectious diseases. The study revealed that although primary school-aged children already know the term “bacteria”, they are generally unable to distinguish between beneficial (“good”) and pathogenic (“bad”) bacteria. It is interesting to note that the term “viruses”—at least in the pre-COVID-19 era—was used much less frequently. While the school children of this age were able to name respiratory and gastrointestinal infections, they were not yet able to differentiate precisely between infections and symptoms.

It is particularly noteworthy that the majority of school children included in this study don’t like to use the sanitary facilities at their school. Moreover, many school children even avoided using the toilets at school, regardless of gender. The reasons given were diverse but predominantly related to the poor cleanliness and general conditions of the sanitary facilities. Our results underscore the importance of continually addressing the topic of school sanitary facilities and they should alert the respective school supervising authorities. Our study emphasizes that health promotion among school children is a participatory and inclusive process that must actively involve not only the institution “school” but also the parents and the children. The results provide the opportunity to highlight the importance of hand hygiene in the semi-public environment, especially in the sanitary facilities of the primary school, which could raise interest in this topic among guardians, school personnel, school authorities and political authorities.

In general, scientific research questions that deal with the target group of children should increasingly involve them and question them directly. Although school children at the primary school level are interested in hygiene- and infection prevention-related issues, there are knowledge gaps that prevent appropriate hygienic behavior in the semi-public environment of sanitary facilities. The ongoing COVID-19 pandemic underlines the importance of sticking to hygienic rules.

## Figures and Tables

**Table 1:** School grades for the sanitary facilities of the primary schools (*n* = 492).

School Grade	Number (Percentage) ^1^ of School Children ^2^		
	Girls (%)	Boys (%)	Total (%)
1 (Very Good)	19 (3.9)	21 (4.3)	40 (8.1)
2 (Good)	55 (11.2)	32 (6.5)	87 (17.7)
3 (Satisfactory)	92 (18.7)	57 (11.6)	149 (30.3)
4 (Sufficient)	57 (11.6)	64 (13.0)	121 (24.6)
5 (Poor)	22 (4.5)	31 (6.3)	53 (10.8)
6 (Deficient)	11 (2.2)	31 (6.3)	42 (8.5)
Total	256 (52.0)	236 (48.0)	492 (100.0)

^1^ Due to rounding to one decimal position, the percentage values do not always add up to 100.0%. ^2^ The total number of school children included in this table is one child lower than the total number of participants due to the absence of one gender specification.

**Table 2:** Frequency distribution in relation to visiting the toilet at school per day.

Frequency to Visit the Toilet at School per Day	Number (Percentage) ^1^ of School Children		
	Girls (%)	Boys (%)	Total (%)
Mostly not at all	112 (22.7)	106 (21.5)	218 (44.2)
1 time	44 (8.9)	44 (8.9)	88 (17.8)
2 times	67 (13.6)	55 (11.2)	122 (24.7)
3 times	22 (4.5)	14 (2.8)	36 (7.3)
More than 3 times	9 (1.8)	14 (2.8)	23 (4.7)
Total	254 (51.5)	233 (47.3)	487 (98.8) ^2^

^1^ Due to rounding to one decimal position, the percentage values do not always add up to 100.0%. ^2^ The missing 1.2% are explained by those school children who did not answer the question.

**Table 3:** Categories and examples of answers to the question “Do you agree with the following sentence: I like to go to the toilet at school. I don’t like to go to the toilet at school, because…”?

Category ^1^	Quantity of Answers ^2^	Exemplary Answer
Uncleanliness of the sanitary facilities	243	“… the toilets are always dirty.” (boy, 9 years old) “…and there is not much cleaning.” (girl, 9 years old) “…there are mostly spiders.” (girl, 9 years old) “…the toilet is a bit disgusting.” (girl, 8 years old)
(Mis-)behavior of classmates	141	“…sometimes there is urine on the toilet seat.” (girl, 9 years old) “…no one washes their hands and then touches the door handle!” (girl, 8 years old) “the toilet seat is sometimes dirty and some forget to flush.” (girl, 9 years old) “…they always look from underneath, when you are sitting on the toilet.” (boy, 10 years old) “…everyone from school goes to the toilet.” (girl, 10 years old) “…some children put themselves on the toilet with street shoes and then the toilet is dirty.” (girl, 9 years old)
General condition of the sanitary facilities (e.g., aeration, temperature, operability of the equipment and lack of hygiene articles)	82	“…it smells in the toilet.” (girl, 10 years old) “…the toilet room is often wet.” (boy, 9 years old) “…it is always cold.” (boy, 9 years old) “…and there is usually no toilet paper.” (girl, 8 years old)
Conflict of interests	18	“…I won’t be able to learn.” (boy, 9 years old) “…If it takes too long time (to go to the toilet), I miss the lesson or don’t get to know some nice things.” (girl, 9 years old)
Lack of need	14	“… I do not need to (use the toilet).” (girl, 9 years old)
Discomfort using the toilet	14	“…it is scary down there.” (boy, 9 years old) “…it’s uncomfortable for me there.” (girl, 9 years old) “…I feel more at ease at home.” (girl, 9 years old)
Awareness of microbial pathogens	8	“…there are a lot of bacteria.” (girl, 9 years old) “…because there can also always be bacteria.” (girl, 8 years old)
Other	7	“…because otherwise we are always get trouble.” (boy, 8 years old)“…it is impolite.” (boy, 9 years old) “…that is no fun.” (boy, 9 years old)

^1^ Two answers were considered as inappropriate because they did not fit into the context of the question. The sum of the answers classified by category is higher than the total number of the school children´s answers because the listing of more than one reason was allowed and some answers could be assigned to several categories. ^2^ Total: 400 answers.

**Table 4:** Results to various statements regarding “I wash my hands at school…”.

Statements (Number of Valid Answers) “I Wash My Hands at School…”	Number (Percentage) ^1^ of Answers			
	Never (%)	Seldom (%)	Often (%)	Always (%)
1. “…when the hands are dirty.” (*n* = 492)	11 (2.2)	51 (10.4)	157 (31.9)	273 (55.5)
2. “…before eating, e.g., bread during the break or lunch.” (*n* = 489)	120 (24.5)	134 (27.4)	119 (24.3)	116 (23.7)
3. “…before I go to the toilet.” (*n* = 485)	366 (75.5)	75 (15.5)	24 (4.9)	20 (4.1)
4. “…after I went to the toilet.” (*n* = 485)	6 (1.2)	21 (4.3)	86 (17.7)	372 (76.7)
5. “…after physical education.” (*n* = 490)	294 (60.0)	139 (28.4)	40 (8.2)	17 (3.5)
6. “…after the break.” (*n* = 485)	173 (35.7)	182 (37.5)	92 (19.0)	38 (7.8)
7. “…when I worked with paint, e.g., watercolor while art lesson.” (*n* = 491)	7 (1.4)	32 (6.5)	96 (19.6)	356 (72.5)
8. “…after sniffing or blowing the nose.” (*n* = 489)	121 (24.7)	140 (28.6)	111 (22.7)	117 (23.9)
9. “…after sneezing.” (*n* = 488)	150 (30.7)	145 (29.7)	114 (23.4)	79 (16.2)

^1^ Due to rounding to one decimal position, the percentage values do not always add up to 100.0%.

**Table 5:** Results to various statements regarding “I wash my hands at home…”.

Statements (Number of Valid Answers) “I Wash My Hands at Home…”	Number (Percentage) ^1^ of Answers			
	Never (%)	Seldom (%)	Often (%)	Always (%)
1. “…before touching food, e.g., when I help to cook at home.” (*n* = 492)	17 (3.5)	31 (6.3)	70 (14.2)	374 (76.0)
2. “…when the hands are dirty.” (*n* = 491)	6 (1.2)	23 (4.7)	100 (20.4)	362 (73.7)
3. “…before lunch.” (*n* = 490)	31 (6.3)	80 (16.3)	119 (24.3)	260 (53.1)
4. “…before I go to the toilet.” (*n* = 488)	342 (70.1)	98 (20.1)	28 (5.7)	20 (4.1)
5. “…after I went to the toilet.” (*n* = 490)	5 (1.0)	26 (5.3)	88 (18.0)	371 (75.7)
6. “…after I played outside.” (*n* = 489)	54 (11.0)	124 (25.4)	165 (33.7)	146 (29.9)
7. “…after sniffing or blowing the nose.” (*n* = 486)	139 (28.6)	153 (31.5)	90 (18.5)	104 (21.4)
8. “…after sneezing.” (*n* = 488)	171 (35.0)	131 (26.8)	102 (20.9)	84 (17.2)
9. “…after I touched an animal, e.g., a dog or a cat.” (*n* = 486)	90 (18.5)	88 (18.1)	112 (23.0)	196 (40.3)
10. “…when I come home.” (*n* = 490)	94 (19.2)	114 (23.3)	115 (23.5)	167 (34.1)
11. “…before going to bed.” (*n* = 491)	130 (26.5)	111 (22.6)	116 (23.6)	134 (27.3)
12. “…after getting up.” (*n* = 488)	137 (28.1)	117 (24.0)	98 (20.1)	136 (27.9)
13. “…after contact with sick people who are ill with cough or sniffles for example.” (*n* = 489)	18 (3.7)	34 (7.0)	95 (19.4)	342 (69.9)
14. “…when I was at the doctor.” (*n* = 493)	87 (17.6)	97 (19.7)	119 (24.1)	190 (38.5)
15. “…when I visited someone in the hospital.” (*n* = 488)	38 (7.8)	54 (11.1)	97 (19.9)	299 (61.3)

^1^ Due to rounding to one decimal position, the percentage values do not always add up to 100.0%.

**Table 6:** Results for the question: How can you protect yourself against disease?

Statements (Number of Valid Answers)	Options	Number (Percentage) of Answers			Significance ^1^ *p*-Value (Chi-Squared Test ^2^)
		Girls (%)	Boys (%)	Total (%)	
1. “I can be vaccinated against specific diseases.” (*n* = 493)	False	11 (4.3)	14 (5.9)	25 (5.1)	*p* = 0.404 (0.698)
	Right	246 (95.7)	222 (94.1)	468 (94.9)	
2. “I touch my face with unwashed hands after going to the toilet.” (*n* = 493)	False	253 (98.4)	227 (96.2)	480 (97.4)	*p=* 0.118 (2.441)
	Right	4 (1.6)	9 (3.8)	13 (2.6)	
3. “I cover an open wound on my body with a plaster or bandage.” (*n* = 490)	False	16 (6.2)	24 (10.3)	40 (8.2)	*p=* 0.100 (2.707)
	Right	241 (93.8)	209 (89.7)	450 (91.8)	
4. “I keep distance from ill people who are suffer e.g., from cough or sniffles.” (*n* = 491)	False	25 (9.8)	27 (11.4)	52 (10.6)	*p=* 0.556 (0.347)
	Right	230 (90.2)	209 (88.6)	439 (89.4)	
5. “I wash my hands with soap.” (*n* = 492)	False	0 (0.0)	3 (1.3)	3 (0.6)	*p=* 0.108 ^2^ /
	Right	257 (100.0)	232 (98.7)	489 (99.4)	
6. “I play a lot on the computer, game console or watch a lot of television.” (*n* = 485)	False	239 (94.1)	173 (74.9)	412 (84.9)	*p* < 0.001 (34.889)
	Right	15 (5.9)	58 (25.1)	73 (15.1)	
7. “I drink from my classmates’ water bottle.” (*n* = 489)	False	247 (97.2)	229 (97.4)	476 (97.3)	*p=* 0.889 (0.019)
	Right	7 (2.8)	6 (2.6)	13 (2.7)	

^1^ *p* <= 0.05 was considered to indicate a significant difference between boys and girls for the respective statement. ^2^ *p*-values from Pearson’s chi-squared test with the exception of statement 5 where the Fisher’s exact test has been applied. No test statistics calculated.

**Table 7:** Indicated human-to-human infections.

Organ (System) Manifestation	Specified Infections	Number of Answers
Respiratory tract infections	Influenza Cold Tonsillitis Bronchitis Pneumonia	77 18 4 3 2
Gastrointestinal tract infections	Gastrointestinal diseases Hepatitis	84 1
Childhood infections	Scarlet Measles Chickenpox Rubella Mumps	37 15 15 2 1
Skin infections	Herpes Hand-foot-and-mouth disease Shingles Warts Scabies Eczema	8 4 4 2 1 1
Central nervous system infections	Meningitis Rabies	1 1
Eye infections	Conjunctivitis	4
Urinary tract infections	Cystitis	1
Other	Ebola Pest	1 1

**Table 8:** Results on the question: Imagine you have cough, sniffles or diarrhea. How can you help to prevent other people from getting your disease?

Statements (Number of Valid Answers)	Options	Number (Percentage) of Answers			Significance *p*-Value (Chi-Squared Test ^1^)
		Girls (%)	Boys (%)	Total (%)	
1. “I will stay at home if possible.” (*n* = 492)	False	7 (2.7)	12 (5.1)	19 (3.9)	*p =* 0.176
	Right	249 (97.3)	224 (94.9)	473 (96.1)	(1.827)
2. “I don’t shake hands with other people.” (*n* = 492)	False	24 (9.3)	22 (9.4)	46 (9.3)	*p =* 0.993
	Right	233 (90.7)	213 (90.6)	446(90.7)	(0.000)
3. “I cough or sneeze in the crook of my arm and not into my hand.” (*n* = 492)	False	9 (3.5)	10 (4.2)	19 (3.9)	*p =* 0.678
	Right	247 (96.5)	226 (95.8)	473 (96.1)	(0.172)
4. “I cough or sneeze into the hand and not in the crook of my arm.” (*n* = 491)	False	241 (94.1)	216 (91.9)	457 (93.1)	*p =* 0.332
	Right	15 (5.9)	19 (8.1)	34 (6.9)	(0.942)
5. “I don’t spit on the floor.” (*n* = 489)	False	33 (12.9)	40 (17.1)	73 (14.9)	*p =* 0.198
	Right	222 (87.1)	194 (82.9)	416 (85.1)	(1.657)
6. “I meet with my friends.” (*n* = 491)	False	235 (92.2)	207 (87.7)	442 (90.0)	*p =* 0.101
	Right	20 (7.8)	29 (12.3)	49 (10.0)	(2.696)
7. “I wash my hands especially thoroughly after using the toilet.” (*n* = 493)	False	3 (1.2)	7 (3.0)	10 (2.0)	*p =* 0.206 ^1^
	Right	254 (98.8)	229 (97.0)	483 (98.0)	/

^1^ *p*-values from Pearson’s chi-squared test with the exception of statement 7 where the Fisher’s exact test has been applied. No test statistics calculated.

## Data Availability

The datasets generated and/or analysed in the current study are not publicly available due to data privacy, but are available from the corresponding author on reasonable request.

## Appendix A

**Table A1:** Detailed information of the participating primary schools.

Municipalities ^1^	Number of Inhabitants ^2^	City and Municipality Type ^3^	Number of Primary Schools in the Municipalities ^4^	Number of Participating Primary Schools from the Municipalities
Altenberge	10,327	Larger small town	2	2
Emsdetten	36,029	Little medium-sized town	6	1
Greven	37,753	Little medium-sized town	5	2
Hörstel	20,344	Little medium-sized town	5	2
Ladbergen	6688	Little small town	1	1
Laer	6744	Little small town	1	1
Lengerich	22,660	Little medium-sized town	4	3
Lienen	8604	Little small town	3	1
Lotte	14,095	Larger small town	3	1
Westerkappeln	11,241	Larger small town	2	1

^1^ With participation of one or more primary schools. ^2^ Source, Landesbetrieb IT.NRW. Available online: https://www.it.nrw/statistik/eckdaten/bevoelkerung-nach-gemeinden-93051 (accessed on 31 December 2019). ^3^ Source, Bundesinstitut für Bau-, Stadt- und Raumforschung. Available online: https://www.bbsr.bund.de/BBSR/DE/forschung/raumbeobachtung/Raumabgrenzungen/deutschland/gemeinden/StadtGemeindetyp/StadtGemeindetyp.html (accessed on 31 December 2019). ^4^ Source, Kreis Steinfurt. Available online: https://www.kreis-steinfurt.de/kv_steinfurt/Kreisverwaltung/Ämter/Amt%20für%20Schule,%20Sport%20und%20Integration/Schulen%20im%20Kreis%20Steinfurt/ (accessed on 31 December 2019).

**Table A2:** Number of classes per school and information about the sanitary facilities.

Primary School ^1^	Total Number of School Children ^2^	Number of Classes	Number of Grade 3 Classes	Number of Sanitary Facilities for School Children (Girls/Boys) ^3^	Total Number of Toilets Seats for School Children Girls/Boys/Total (%) ^4^	Total Number of Urinals for School Children (Only Boys)
01	268	12	3	4 (2/2)	7/4/11 (4.1)	10
02	261	11	2	8 (4/4)	11/7/18 (6.9)	14
03	157	8	2	6 (3/3)	8/6/14 (8.9)	12
04	340	14	3	5 ^5^	13/8/21 (6.2)	7
05	267	11	3	2 (1/1)	7/5/12 (4.5)	10
06	104	4	1	2 (1/1)	3/4/7 (6.7)	2
07	103	4	1	2 (1/1)	4/2/6 (5.8)	4
08	161	8	2	2 (1/1)	4/5/9 (5.6)	5
09	94	4	1	2 (1/1)	4/3/7 (7.4)	4
10	202	8	2	2 (1/1)	4/3/7 (3.5)	3
11	240	11	3	4 (2/2)	10/7/17 (7.1)	11
12	192	8	2	2 (1/1)	6/6/12 (6.2)	2
13	226	12	3	2 (1/1)	14/7/21 (9.3)	7

^1^ Pseudonymized. ^2^ It should be noted that the total number of school children may fluctuate slightly during the school year. ^3^ Without sanitary facilities for children with disabilities. ^4^ Percentage of toilets seats in relation to the total number of school children. ^5^ Consisting of one sanitary facility for girls, one sanitary facility for boys and three sanitary facilities used by both genders.

**Table A3:** Characteristics of the school children (*n* = 492).

Age in Years	Number (Percentage) ^1^ of School Children		
	Girls	Boys	Total
8	58 (11.8%)	45 (9.1%)	103 (20.9%)
9	178 (36.2%)	168 (34.1%)	346 (70.3%)
10	19 (3.9%)	18 (3.7%)	37 (7.5%)
11	2 (0.4%)	4 (0.8%)	6 (1.2%)
Total	257 (52.2%)	235 (47.8%)	492 (100.0%)

^1^ Due to rounding to one decimal position, the percentage values do not always add up to 100.
